# Genetic resources and breeding of maize for *Striga* resistance: a review

**DOI:** 10.3389/fpls.2023.1163785

**Published:** 2023-05-10

**Authors:** Emeline Nanou Dossa, Hussein Shimelis, Emmanuel Mrema, Admire Tichafa Isaac Shayanowako, Mark Laing

**Affiliations:** ^1^ School of Agricultural, Earth and Environmental Sciences, University of KwaZulu-Natal, Pietermaritzburg, South Africa; ^2^ Tanzania Agricultural Research Institute, Tumbi Center, Tabora, Tanzania

**Keywords:** doubled haploid, genetic resources, gene editing, genomic resources, maize breeding, quantitative traits loci, *Striga* species

## Abstract

The potential yield of maize (*Zea mays* L.) and other major crops is curtailed by several biotic, abiotic, and socio-economic constraints. Parasitic weeds, *Striga* spp., are major constraints to cereal and legume crop production in sub-Saharan Africa (SSA). Yield losses reaching 100% are reported in maize under severe *Striga* infestation. Breeding for *Striga* resistance has been shown to be the most economical, feasible, and sustainable approach for resource-poor farmers and for being environmentally friendly. Knowledge of the genetic and genomic resources and components of *Striga* resistance is vital to guide genetic analysis and precision breeding of maize varieties with desirable product profiles under *Striga* infestation. This review aims to present the genetic and genomic resources, research progress, and opportunities in the genetic analysis of *Striga* resistance and yield components in maize for breeding. The paper outlines the vital genetic resources of maize for *Striga* resistance, including landraces, wild relatives, mutants, and synthetic varieties, followed by breeding technologies and genomic resources. Integrating conventional breeding, mutation breeding, and genomic-assisted breeding [i.e., marker-assisted selection, quantitative trait loci (QTL) analysis, next-generation sequencing, and genome editing] will enhance genetic gains in *Striga* resistance breeding programs. This review may guide new variety designs for *Striga*-resistance and desirable product profiles in maize.

## 1 Introduction

Maize (*Zea mays* L., 2n = 2x = 20) is a staple cereal crop after rice and wheat worldwide, accounting for 30–70% of the total caloric consumption (Kamara et al., 2005; Shiferaw et al., 2011; Yacoubou et al., 2021b). The annual global production of maize was estimated at 1,210,235,135.14 tons in 2021 (FAOSTAT, 2022). The main maize producers are the United States of America (592,356,330.09 tons), China (272,762,124 tons), Brazil (88,461,943 tons), Argentina (60,525,80 tons), Ukraine (42,109,850 tons), India (31,650,000 tons), and Mexico (27,503,477.82 tons), accounting for about 66% of the global production per annum (FAOSTAT, 2022). In Africa, annual maize production was estimated at 96,637,314.23 million tons in 2021, representing 7.98% of the world’s production (FAOSTAT, 2022). South Africa is the largest maize producer in Africa, with an estimated annual production of 16,870,705 million tons, followed by Nigeria (12,745,000 tons), Ethiopia (10,722,000 tons), Egypt (7,500,000 tons), and Kenya (3,303,000 tons) (FAO, 2022).

In sub-Saharan Africa (SSA), 14 countries have the highest per capita consumption of maize (Ranum et al., 2014). For instance, in Benin, the mean per capita consumption of maize per annum is 85 kg (Hongbete et al., 2017). Maize is produced in all agroecological zones in Africa on smallholder landholding varying between 0.5 and 2 hectares (ha) (Achigan-Dako et al., 2014). SSA has the lowest maize yields globally, estimated at 3 tons/ha (FAO, 2022), compared with global mean yields of 5 to 10 tons ha^-1^ (FAO, 2022). The low productivity of maize and major crops in SSA is attributable to an array of production constraints, including biotic factors (e.g., parasitic and competitive weeds, field and storage insect pests, and pre- and post-harvest diseases) (Cairns et al., 2013; Rachidatou et al., 2018; Yacoubou et al., 2021a), and abiotic factors including heat and drought stresses (Cairns et al., 2013; Ali et al., 2015). Heat and drought stress, coupled with low soil fertility, are some of the common abiotic challenges affecting maize productivity throughout the region (Badu-Apraku and Fakorede, 2017; Lobulu et al., 2019), predisposing major cereal and legume crops to parasitic weeds of the genus *Striga*.

**Figure 1:**
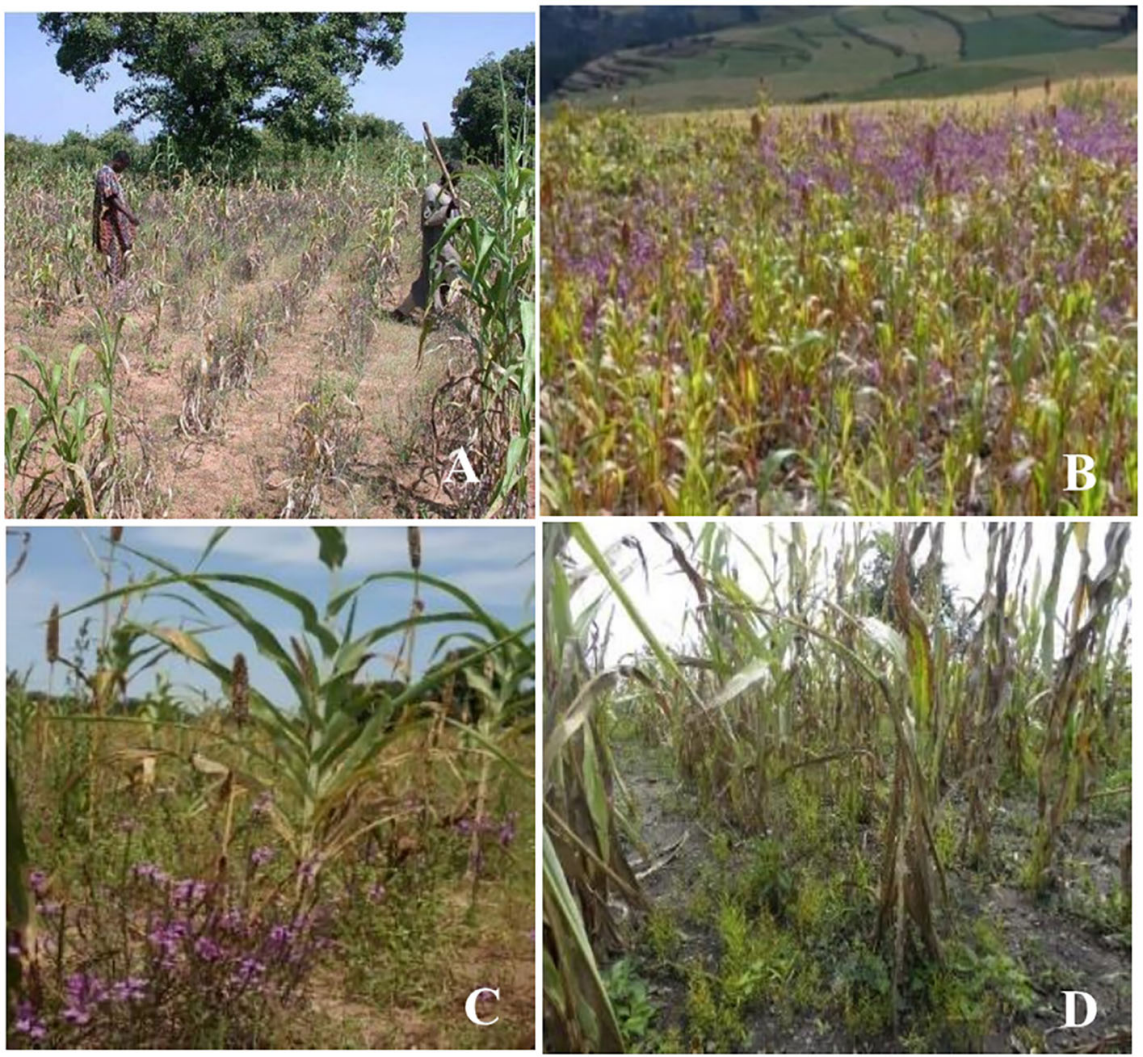
*Striga hermonthica* infested-maize in Benin **(A)**, sorghum in Ethiopia **(B)**, pearl millet in Burkina-Faso **(C)**, and *S. asiatica* infested maize in Tanzania **(D)**. (Photo A supplied by Dr. Fen Beed, B by H Shimelis, C by S Al-Babili, and D by E Mrema).

Forty *Striga* species have been reported worldwide (Gethi and Smith, 2004; Reda and Verkleij, 2004). Among these, 33 species have been reported in Africa and 11 affect major grain crops (Gethi and Smith, 2004; Ejeta et al., 2007). *Striga hermonthica* (Del.) Benth and *Striga asiatica* (L.) Kuntze are the most economically important in cereal production systems. *Striga hermonthica* is present in most SSA regions, affecting Western, Central, and Eastern Africa (**Figure 1A, C, and D**), while *S. asiatica* (**Figure 1B**) is predominant in Southern Africa (Ejeta, 2007; Parker, 2012; Shayanowako et al., 2018a).

Unlike sorghum, which has co-evolved with *Striga* weed in Africa, maize is exceptionally susceptible to the parasite, particularly in marginal and low input production environments (Adewale et al., 2020; Badu-Apraku et al., 2020a; Shayanowako et al., 2020). Maize yield losses exceeding 60% are common under *Striga* infestation (Jamil et al., 2012; Lobulu et al., 2019). Global *Striga*-infested area in SSA is estimated to be 2.4 million ha leading to a yield loss of 1.6 tons per year valued at US$ 383 million (Woomer et al., 2008). Methods to control *Striga* include cultural practices, chemical herbicides, and biological agents. However, these are often unavailable or inadequate (Mrema et al., 2017), and farmers often resort to hand hoeing, which is ineffective because most of the damage to the maize plant occurs before the parasitic plants emerge (Ejeta, 2007; Stanley et al., 2021). There is little use of selective herbicides in the region because of the high cost and the complex cropping systems (e.g., intercropping cereals and legumes) (Hearne, 2009). The use of host resistance against *Striga* parasitism is widely favored because of its cost effectiveness, safety, and practicality, making it readily deployable in low-input maize production environments (Badu-Apraku et al., 2016; Adu et al., 2019). Breeders strive to develop maize genotypes that are either (i) resistant, inhibiting or allowing few *Striga* seeds to germinate and attach onto the host roots through low production of strigolactones (SL), root barriers for incompatibility, and hypersensitive and antibiosis reactions toxic to the parasite, or (ii) tolerant by being insensitive to high levels of parasitic attachments with minimum yield loss.

Presently, three component traits are used to rate the performance of a genotype under *Striga* infestation: (1) *Striga* emergence count, (2) *Striga* damage rating, and (3) crop grain yield (GY) performance. *Striga* emergence count determines the extent of suppression of *Striga* attachments, which reflects active host resistance. *Striga* damage ratings and GY response of the host are measures of tolerance used to rate the degree to which a maize plant can withstand the phytotoxic effects of *Striga* among genotypes that support many parasitic attachments. Ideally, *Striga*-resistant maize would be attractive in that it would also cause a reduction in the *Striga* seed bank compared with tolerance (Rodenburg and Bastiaans, 2011). However, *Striga* resistance has quantitative inheritance and is controlled by multiple minor genes with additive gene action. Durable resistance is yet to be reported in maize. Highly tolerant genotypes allow parasitic attachments of *Striga* plants resulting in the seed set and dispersal of the weed (Yacoubou et al., 2021b). Furthermore, resistance genes may be effective only in a specific region or agroecologies due to genotype by environment interaction effect (Oswald and Ransom, 2002).

Hindrances to developing maize cultivars with durable resistance include several aspects, such as a focus on a narrow spectrum of resistance traits during breeding. For example, most of the previous breeding programs involving field and control environment screening did not consider the SL biosynthesis effect, which is the basis of *Striga*-resistant traits. It has been reported that SL analogs induce suicidal germination of *Striga* spp. seed in soil (Kountche et al., 2019). Jamil et al. (2022) evaluated the efficacy of three potent SL analogs under laboratory, greenhouse, and farmers’ field conditions. The authors confirmed that the selected SL analogs appear to make promising candidates under field conditions that led to 43 and 60% reduction of *Striga* emergence in pearl millet and sorghum, respectively. Li et al. (2023) identified SL, zealactol, and zealactonoic acid compounds. The reported components stimulate less *Striga* germination than the earlier reported SL and zealactone from maize. The authors demonstrated that maize genotypes primarily producing zealactol suffered less *Striga* damage than genotypes with higher zealactone content. Genes controlling *Striga* resistance might range from major genes, that is, those responsible for the production of SL (Lopez-Raez et al., 2009) to quantitative traits loci (QTLs) influencing the expression of resistance in the field (Amusan, 2010; Badu-Apraku et al., 2020a). Field resistance to *Striga* determines the ultimate purpose of varietal improvement.

The application of modern genomic tools has been effective in dissecting complex traits conditioning biotic and abiotic tolerance (Gedil and Menkir, 2019). Hence, developing and applying genomic tools for *Striga* resistance will be critical in accelerating breeding for *Striga* resistance. Genomic-assisted breeding complements the traditional approaches in integrating resistance to *Striga* and other quantitatively inherited traits of importance to food security (Michel et al., 2017). *Striga* tolerance is highly effective in maize, while it may not provide complete protection due to partial resistance (Shayanowako et al., 2018b). Additionally, environmental factors such as poor soil fertility, erratic rainfall, and high temperatures favor the high fecundity of *Striga* (Chidiebere et al., 2015). Hence, there is a need to integrate host tolerance and other *Striga* management methods to reduce the damage caused by the parasite.

The development of *Striga*-resistant and market-preferred maize cultivars is among the primary goals of public and private maize breeders. The design of maize ideotypes for *Striga* resistance/tolerance and desirable product profiles depends on integrating multiple winning and essential traits based on host-parasite interaction. Hence, exploiting the genetic variation and defense mechanisms present in the host against the parasites may accelerate the adoption of novel breeding approaches in maize. In light of the above background, this review aims to present the genetic and genomic resources, research progress, and opportunities in the genetic analysis of *Striga* resistance and yield components in maize. Information presented in this paper may guide new variety designs in maize for *Striga*-resistance and desirable product profiles.

## 2 Genetic resources of maize for *Striga* resistance

A successful genetic improvement for *Striga* resistance depends on the host’s natural or induced genetic variation. Several genetic sources of *Striga* resistance have been reported in maize (Gurney et al., 2002; Rich and Ejeta, 2008) and other cereal crops, including sorghum (Gurney et al., 2002; Rich et al., 2004), rice, and pearl millet (Wilson et al., 2000). **Table 1** presents some of their genetic sources with their unique resistant traits. The summary presents variability of *Striga* resistance sources, including landraces, cultivars, wild relatives, breeding lines, and single cross hybrids for each of the crops. It also portrays *Striga* resistance traits such as a low haustorium initiation, *Striga* germination, *Striga* attachment, a hypersensitive reaction by the host, and parasitic mortality.

**Table 1:** Some genetic sources of *Striga* resistance in maize, sorghum, pearl millet, and rice.

Crops	Type of variety	Name or designation	Striga reaction	Unique traits	Country and reporting organization	References
**Maize**	Wild relative	*Tripsacum dactyloides*	Pre-attachment resistant	Inhibition of haustorial development	IITA, Nigeria	Gurney et al. (2003)
		*Zea diploperenis*	Post-attachment resistant	Barrier development after haustorial development		Amusan et al. (2008)
	Line	ZD05	Post-attachment resistant	Low level of *Striga* attachment and high mortality of attached parasites		
		TZdEEI 7	Post-attachment resistant	Barrier development after haustorial development		Shaibu et al. (2021)
		TZEEI 63				
		TZdEEI 1				
	Landraces	CRIC 51	Pre-attachment resistant	Low level of *Striga* germination	CIMMYT, Kenya IITA, Nigeria, KARI, Kenya	Karaya et al. (2012)
		VERA 217				
		CUBA T-31				
		BRAZ 1758				
		BRAZ 1279				
		CRIC 51				
		Mochore	Pre-attachment resistant	Low level of *Striga* germination	ICIPE, Kenya	Midega et al. (2016)
		Nyamula				
		Sefensi				
		Jowi				
**Sorghum**	Wild relatives	*Sorghum versicolor*	Post-attachment resistant	Hypersensitivity	IACR-Long Ashton Research Station	Haussmann et al. (2000)
		*Sorghum drummondii*	Pre-attachment resistant	Low haustorium initiation		Ramaiah (1986)
	Lines	SRN 39	Post-attachment resistant	Low production of the germination stimulant	ICRISAT, Burkina-Faso	
		IS 9830	Post-attachment resistant	Low production of the germination stimulant		
		IS 15401	Post-attachment resistant	Low production of the germination stimulant		
		SAR 16	Post-attachment resistant	Low production of the germination stimulant, hypersensitivity		
		SAR 19	Post-attachment resistant	Low production of the germination stimulant, hypersensitivity		
		SAR 33	Post-attachment resistant	Low production of the germination stimulant, hypersensitivity		
	Cultivars	N 13	Post-attachment resistant	Mechanical barriers, antibiosis	ICRISAT, Mali	Gurney et al. (2002); Haussmann et al. (2000)
		Framida	Post-attachment resistant	Mechanical barriers	ICRISAT, Mali	
**Pearl millet**	Wild accessions	*PS 202*, PS 637, PS 639, PS 727	Pre-attachment resistant	Low level of *Striga* attachement	ICRISAT, Mali	Wilson et al. (2000)
	Landraces	M141, M239, M029, M197, M017, and KBH	Pre-attachment resistant	Lower level of *Striga* attachment, lower downy mildew incidence, higher panicle yield	IRD, France ICRISAT, Niger	Kountche et al. (2013)
**Rice**	Cultivars	Nipponbare	Post-attachment resistant	Absence of parasite–host xylem–xylem connections	IRRI, Philippines	Gurney et al. (2006)

IRD, Institute for Research Development/France; ICRISAT, International Crops Research Institute for the Semi-Arid Tropics/India; IRRI, International Rice Research Institute/Philippines; IACR, Institute for Arable Crops Research/India; IITA, International Institute of Tropical Agriculture/Nigeria; KARI, Kenya Agricultural Research Institute; ICIPE, International Centre of Insect Physiology and Ecology/Kenya.

**Table 2** presents gene banks and databases of maize genotypes with *Striga* resistance. *Striga*-resistant varieties are curated at International Institute of Tropical Agriculture (IITA/)Nigeria, CIMMYT/Zimbabwe, KARI/Kenya, NARLWEYO Seed company/Uganda, and Meru Agro seed company/Tanzania. These gene banks and databases serve to acquire new plant materials for *Striga* resistance breeding and genetic conservation for medium and long-term use (Smale and Jamora, 2020).

**Table 2:** Important gene banks and databases of maize as a source of germplasm for *Striga* resistance.

Gene bank	Country	Reference
**International Institute of Tropical Agriculture (IITA)**	Nigeria	Badu-Apraku and Yallou (2009)
**International Maize and Wheat Improvement Center (CIMMYT)**	Zimbabwe	Dhliwayo et al. (2021)
**Kenya Agricultural Research Institute (KARI)**	Kenya	Okora et al. (2006)
**NALWEYO Seed Company (NASECO)**	Uganda	Tugendhat (2017)
**Meru Agro seed company**	Tanzania	Kanampiu et al. (2005)

### 2.1 *Striga* resistance/tolerance mechanisms

Several *Striga* resistance mechanisms are reported. The mechanisms act either before (pre-attachment) or after physical contact with the host (post-attachment). In the case of pre-attachment resistance, the host produces low levels of SL, below which the *Striga* receptors perceive the germination stimulants insensitive to SL, inducing less parasitic germination (Ejeta and Gressel, 2007).

Host resistance has been identified in an opened-pollinated maize variety (OPV) KSTP 94 in western Kenya, where *S. hermonthica* is dominant (Yoneyama et al., 2015). Post-attachment resistance occurs after *Striga* attachment to the host. Maize inbred line ZD05 and an open-pollinated variety KSTP 94, are some of the sources of resistance identified. ZD05 exhibits low levels of *Striga* attachment and high mortality of attached parasites compared with the susceptible inbred line 5057 (Amusan et al., 2008). KSTP 94 develops low numbers of *Striga* attachments and biomass compared with the susceptible inbred line CML 144 (Mutinda et al., 2018). The two maize varieties have different resistance mechanisms, which facilitate the introgression of *Striga* resistance genes into farmer-preferred and susceptible germplasm.

### 2.2 Genetic variation as a source of *Striga* resistance/tolerance in maize

The genetic variation between germplasm resources within plant species is useful for the development of improved varieties with desirable traits, including *Striga* resistance (Holme et al., 2019; Latpate, 2019). This enables variety design with market-preferred traits, resistance/tolerance against biotic and abiotic factors, and adaptation to diverse agro-ecological zones.

**Table 3** presents some modern maize genotypes reported to be *Striga* resistant/tolerant and possessing good agronomic traits. Lower *Striga* damage rating and lower *Striga* emergence count are the main components of *Striga* resistance in host crops. Genetic variation existing in gene pools may occur naturally in landraces, elite varieties, cultivated and obsolete cultivars, or artificially created through mutation breeding or genetic engineering (Hawkes, 1991). Major sources of *Striga* resistance gene in maize have been derived from wild relatives, landraces, synthetics, composites, and elite breeding lines (Taba, 1995; Sachs, 2009) as described in the following section. The novel genetic sources are ideal for use in pre-breeding and breeding programs for improving *Striga* resistance and agronomic traits.

**Table 3:** Some modern maize genotypes reported to be *Striga* resistant and with good agronomic traits.

Type of variety	Name or designation	*Striga* reaction	Pedigree	Country and reporting organization	References
**Inbred lines**	TZEI 2	Resistant	TZE-W Pop × 1368 STR S7 Inb 2	International Institute of Tropical Agriculture, Nigeria	Akinwale et al. (2013)
	TZEI 83	Tolerant	TZE-W Pop × 1368 STR S7 Inb 8		
	TZEI 124	Tolerant	TZE-Y Pop STR C0S6 Inb 3 1-3		
	TZEI 136	Resistant	TZE-Y Pop STR C0S6 Inb 21 1–3		
	TZEI 14	Resistant	TZE Comp 5 Y C6S6 Inb 21		
	TZEI 23	Resistant	TZE-Y Pop STR C0S6 Inb 62 2-3		
	TZEI 81	Tolerant	TZE-W Pop × 1368 STR S7 Inb 5		
	TZISTR1108	Resistant	Z. Diplo. BC4-472-2-1-1-2-B-1-B	International Institute of Tropical Agriculture, Nigeria	Menkir et al. (2006)
	TZSTRI107	Resistant	(ACRSYN-W-S2-173- B*4/TZLCompIC4S1-37-1-B*4)-4- B*4	International Maize and Wheat Improvement Center, Zimbabwe	Gasura et al. (2019)
	TZISTR1161	Resistant	(ACRSYN-W-S2-173- B*4/TZLCompIC4S1-37-5-BBB)-3- B*4		
	TZISTR1224	Resistant	(ACR97SYN-Y-S1-79- B*4/ACR97TZLComp1-YS155-4-1- 3-B*4)-9-1-BB-B		
	TZISTR1248	Resistant	(ACR97TZLComp1-YS155-4-1-3- B*4/ACR97SYN-Y-S1-76-B*4)-32- 1-BB-B		
**OPVs**	ACR94TZE, COMP5-W	Resistant	–	International Institute of Tropical Agriculture, Nigeria	Menkir and Kling (2007)
	ACR97 TZL CMP1-W	Resistant			
	TZEE-Y Pop STR	Resistant			
	2004 TZEE-Y Pop STR C4	Resistant			
	TZEE-W Pop STR QPM C0	Resistant			
	TZEE-W Pop STR BC2 C0 TZEE-W	Resistant			
	STR 107 BC1 TZEE-W STR 107	Resistant			
	BC1 TZEE-W STR 107 BC1	Resistant			
	KSTP 94, STR-VE-216	Resistant	–	Kenya Agricultural & Livestock Research Organization, Kenya	Mutinda et al. (2018)
**Single cross hybrids**	TZISTR1162x×TZISTR1198	Resistant		National Crops Resources Research Institute, Uganda	Simon et al. (2018)
	TZISTR1199×TZISTR1181	Resistant			
	TZISTR1192 × 1368STR	Resistant			
	TZEIOR 57 × TZEI 10	Resistant		International Institute of Tropical Agriculture, Nigeria	Kim (1994), Konate et al. (2017)
	TZEIOR 127 × TZEI 10	Resistant			
	8322-13	Resistant			
	8321-18	Resistant			
	TZEIOR 57 × TZEIOR 108	Resistant			
	TZEIOR 57 × TZEIOR 127	Resistant			
	TZEIOR 13 × TZEIOR 59	Resistant			
	TZISTR1162x×TZISTR1198	Resistant			
**Synthetics**	2008 SYN EE-W DT STR	Resistant	–	International Institute of Tropical Agriculture, Nigeria	Badu-Apraku et al. (2010), Badu-Apraku et al. (2016), (Oluwaranti et al., 2020)
	2008 TZEE-W STR	Tolerant			
	2009 TZEE-OR2 STR	Resistant			
	FERKE TZEE-W STR	Resistant			
	TZEE-W DT C0 STR C5	Resistant			
	2012 TZEE-W DT STR C5	Resistant			
	Syn TZB STR	Resistant	–	International Institute of Tropical Agriculture, Nigeria	Kim et al. (1998), Aliyu et al. (2004)
	STR Syn-W	Resistant			
	STR Syn-Y	Resistant			
	STR SynY/W	Resistant			
	Syn TZSR-Y-I STR	Resistant			
	TZB-Saminaka STR	Resistant			
	EV Tuxp. S6quia STR TZDT-SR-STR-2	Resistant			
	TZDT-SR-STR-3	Resistant			
	EV 49 SR-STR	Resistant			
	Pool 16 DT-SR-STR	Resistant			
	Suwan 1 SR-STR	Resistant			
	TZ Syn-W STR	Resistant			
	Perennial STR-SR	Resistant			
	TZ Syn-Y STR	Resistant			
**Composites**	TZE Comp. 5	Resistant	–		
	Act 93 TZL Comp. I	Resistant			
	TZL Comp. 1	Resistant			
	Acr 92 TZE Comp. 5	Resistant			
	ACR97 TZL COMP1-W	Tolerant			

#### 2.2.1 Landraces

Landraces are dynamic populations of cultivated crop species with a historical origin and distinct identity, often genetically diverse, locally adapted, and derived through a set of farmers’ practices and knowledge of seed selection and field management (Azeez et al., 2018). Landraces or farmers’ varieties serve as genetic resources that can provide essential characteristics such as resistance to diseases and pests, grain quality, and contribute to extending the genetic base of modern cultivars. The International Plant Genetic Resources Institute (IPGRI) maize landraces represent a genetic reservoir for yield improvement in maize (wumasi et al., 2017). The 196 maize landraces reported by Nelimor et al. (2020) represent gene pools from Burkina-Faso, Ghana, and Togo, for specific agronomic traits including GY, stay green trait, number of ear per plant, ear length, and number of rows per ear. Other maize landraces were previously reported by Meseka et al. (2013) from the northern Guinea savanna and Sudan savanna in West and Central Africa and have shown some drought-adaptive traits.

Some maize landraces reported with *Striga* resistance traits are presented in **Table 1**. Despite the paucity of information on *Striga* resistance genes in landraces, genotypes with marked tolerance have been identified (Kim et al., 1999). In a study by Karaya et al. (2012), 420 landraces triggered a lower level of *Striga* germination than commercial checks. The genetic composition of these maize landraces is yet to be confirmed. Genes causing lower levels of *S. hermonthica* emergence in both pot and field trials have been reported in Kenya (Midega et al., 2016). Germplasms with various levels of *Striga* resistance were reported in Western and Eastern Africa (Amusan et al., 2008; Rich and Ejeta, 2008; Badu-Apraku and Yallou, 2009; Mutinda et al., 2018).

#### 2.2.2 Synthetics and composite varieties

Significant progress has been achieved in identifying maize genotypes with *Striga* resistance. Synthetic varieties have greatly improved breeding for *Striga* resistance in maize. Synthetic varieties are random mating populations produced by crossing a group of inbreds with superior general combining ability (Osei et al., 2014). Their prime advantage is that farmers can save their seeds for the next crop cycle. Reportedly, the synthetic population developed by the IITA from 1994 to 1998 with various *Striga* resistance levels is a major source of *Striga* resistance in maize (Kim et al., 1998). The IITA-West and Central Africa programs developed other sources of resistant maize germplasm through the Collaborative Maize Research Network (WECAMAN) (Badu-Apraku et al., 2007b). The germplasm has been used as a source of resistance genes in developing maize genotypes such as 2008 TZEE-W STR, 2009 TZEE-OR2 STR, FERKE TZEE-W STR, TZEE-W DT C0 STR C5, 2012 TZEE-W DT STR C5, and 2008 SYN EE-W DT STR, which produces a high GY under *Striga* infestation (Badu-Apraku et al., 2010b; Badu-Apraku et al., 2016; Oluwaranti et al., 2020). The established resistant genotypes can serve as checks in breeding programs for *Striga* resistance assessment (Badu-Apraku et al., 2008; Menkir et al., 2010). Genetically diverse *S. hermonthica-*resistant inbred lines have been developed by the IITA through trait introgression from resistant germplasm, including some of the synthetics (Gasura et al., 2019).

Other important sources of *Striga* resistance are composites, which are varieties developed by mixing the seeds of phenotypically promising lines. Composites are maintained through open pollination. Open-pollinated varieties (OPVs) are developed through random pollination and seeds from the best-looking individuals are harvested from a heterogeneous population. Kim (1996) reported that composites developed by IITA in 1994 provided a broad genetic pool for resistance breeding against S. *hermonthica.* These composites varieties has been successfully introduced, leading to the development of resistant inbred lines, hybrids, and synthetics (Badu-Apraku et al., 2006; Badu-Apraku et al., 2009; Gasura et al., 2021). Furthermore, OPVs, synthetics, single-cross hybrids, and composites from KARI (now KALRO)/Kenya, and the National Crops Resources Research Institute/Uganda (**Table 3**) are important sources of *Striga* resistance.

#### 2.2.3 Mutant selections

Induced mutagenesis causes a target organism’s deoxyribonucleic acid (DNA) to change, resulting in one or more gene mutations. This will enable the creation of new valuable traits that can be introgressed in well-adapted cultivars (Kozjak and Meglič, 2012). Mutational events can be permanent and heritable genetic changes resulting in phenotypic variation (Ahloowalia and Maluszynski, 2001). However, spontaneous mutation rates in higher plants are low at 10^−6^ to 10^−7^ per locus (Kovalchuk et al., 2000; Jiang and Ramachandran, 2010). Thus induced mutagenesis is an important strategy to increase mutation frequencies (Maluszynski et al., 2000). Mutagenesis has already been used to improve many useful traits in plants (Maluszynski et al., 2000; Wanga et al., 2020).

Selection of mutants for *Striga* resistance has been reported in sorghum. The inheritance of low *Striga* germination stimulant activity was conditioned through a mutant allele expressed in homozygous recessive individuals (Gobena et al., 2017). However, seed mutagenesis of maize using acetolactate synthase inhibiting herbicide (imazapyr), dressed as a drench or as a coating led to the development of herbicide-resistance (Kanampiu et al., 2001; Dan et al., 2017). This enabled an effective, inexpensive, and productive measure to control *Striga*, with immediate benefit to farmers (Paul and Canon, 2008; Babatima et al., 2011). Kiruki et al. (2006) reported that azide-based mutagenesis rendered new mutants that suppressed *S. hermonthica* seed germination and parasitism in maize varieties leading to the conversion of *Striga*-susceptible to *Striga*-resistant maize mutants. These findings confirm that, although natural mutations are known to induce resistance to *Striga*, the process can also be accelerated in the laboratory using azide-based mutagenesis (Kiruki et al., 2006).

#### 2.2.4 Wild relatives

A major limiting factor in breeding maize for yield and yield components and resistance to parasitic weeds, insect pests, and diseases is the narrow base of the genetic diversity of domesticated maize varieties. Wild relatives of maize and their progenitors are reportedly major sources of *Striga* resistance. Teosinte (*Zea diploperennis*), and eastern gamagrass (*Tripsacum dactyloides* L.) have been used in breeding programs as sources of *Striga* resistance (Abdoul-Raouf et al., 2017). The inbred line ZD05 selected for its field resistance to *S. hermonthica* acquired genes from *Z. diploperennis* (Amusan et al., 2008). This breeding line has been useful in developing new ideotypes with economic traits and *Striga* resistance by the IITA/Nigeria in collaboration with the National Agricultural Research System (Diskin et al., 2008). Gurney et al. (2003) evaluated the susceptibility of *T. dactyloides* and a hybrid from *T. dactyloides* and *Z. mays.* The authors found that *T. dactyloides* produces a signal that inhibits *S. hermonthica* haustorial development. This trait could be introgressed into elite maize genotypes for their resistance to *Striga*.

**Figure 2:**
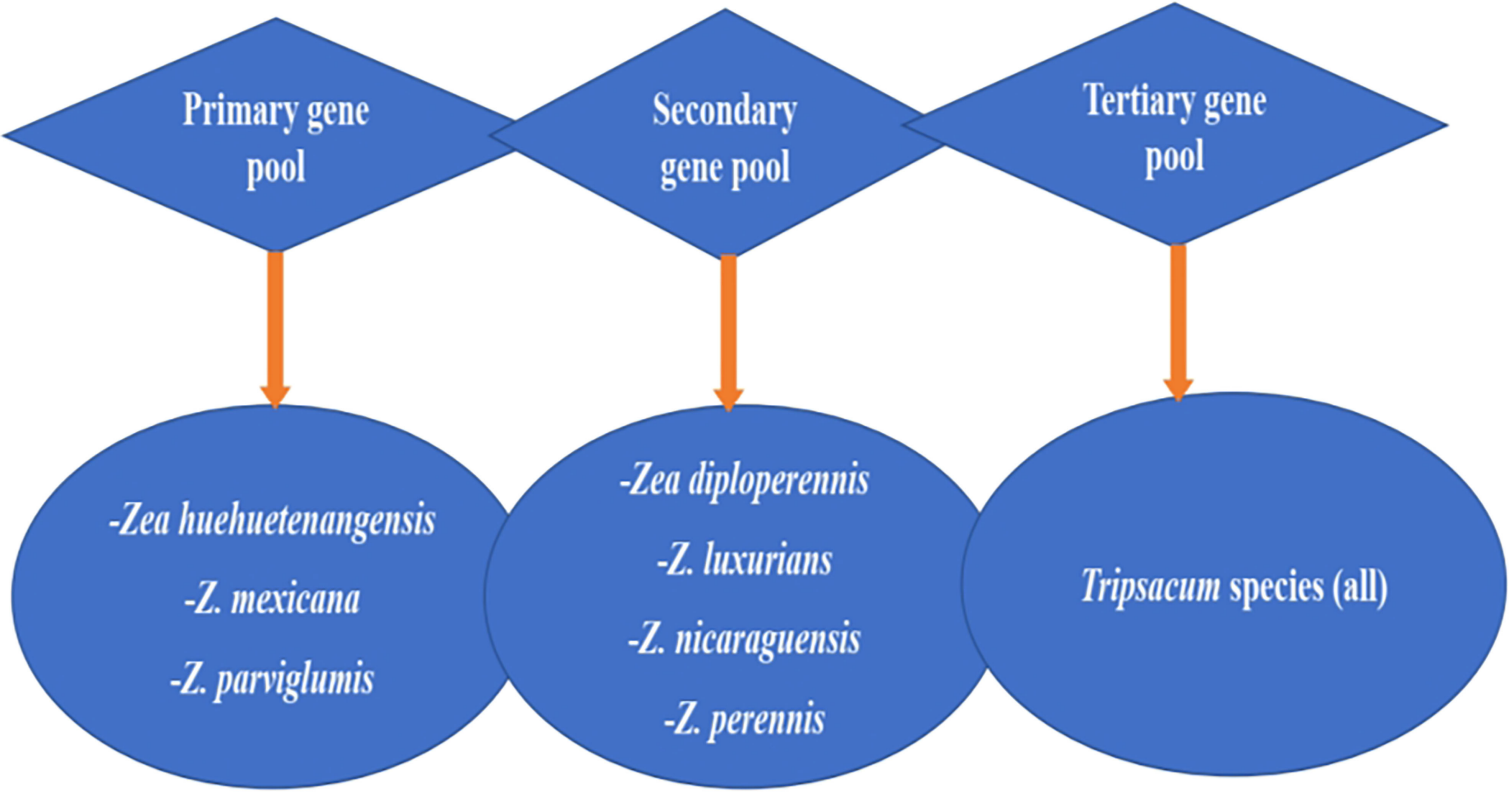
Gene pool classification of maize’s wild relatives. Adapted from Wani et al., 2022.

**Figure 2** presents maize’s wild relatives and the gene pool classified into three groups: primary gene pool, secondary gene pool, and tertiary gene pool, each with different wild genotypes. The primary gene pool includes *Zea huehuetenangensis*, *Z. mexicana*, and *Z.parviglumis*, while the secondary gene pool are *Z. diploperennis*, *Z. luxurians*, *Z. nicaraguensis*, and *Z. perennis.* The tertiary gene pool consists of all the species of the genus *Tripsacum*. These show that there is still more useful genetic diversity available in wild species that could enhance *Striga* resistance in modern maize (Wani et al., 2022). These gene pools could be utilized in modern maize breeding for *Striga* resistance through detecting and transferring useful alleles using hybridization and genetic engineering approaches.

## 3 Genetic gain in breeding maize for *Striga* resistance or tolerance

**Table 4** presents the rates of yield gains realized in *Striga* resistance breeding programs in maize. Information on the genetic progress in crop traits and yield improvement is helpful for direct production or trait integration. Also, genetic modifications and the use of breeding methodologies and strategies are dependent on yield gains (Badu-Apraku et al., 2013). The IITA has recorded a significant genetic gain in maize through released varieties possessing *Striga* resistance (Kamara et al., 2005; Badu-Apraku et al., 2014; Badu-Apraku et al., 2016). GY of the *Striga-*resistant and -tolerant maize varieties under *Striga* infestation enabled measuring the breeding progress (Badu-Apraku et al., 2013; Ifie et al., 2015). The increase in the genetic gain confirms the substantial progress in breeding for high-yielding and *Striga* resistance.

**Table 4:** Rates of yield gains realized in *Striga* resistance in maize breeding program under *Striga*-infested environments.

Year	Yield gain (kg/ha)	Type of variety	References
**1970–1999**	0.41% (2555 kg/ha^−1^ year^−1^)	Opened pollinated varieties	Kamara et al. (2005)
**1982–2010**	3.17% (93.69 kg/ha^−1^ year^−1^)	Hybrids	Menkir and Meseka (2019)
**1988–2010**	1.59% (30 kg/ha^−1^ year^−1^)	Opened pollinated varieties	Badu-Apraku et al. (2014)
**1995–2012**	2.56% (42kg/ha^−1^ year^−1^)	Opened pollinated varieties	Badu-Apraku et al. (2016)
**1995–2012**	2.72% yr^-1^ (44 kg ha^−1^ year^−1^)	Opened pollinated varieties	Badu‐Apraku et al. (2017)
**2008–2015**	4.82% (101 kg ha^−1^ year^−1^)	Hybrids	Badu-Apraku et al. (2020b)
**2014–2017**	16.9% cycle^−1^ (498 kg ha^−1^ cycle^−1^)	Opened pollinated varieties	Badu-Apraku et al. (2019)

## 4 Breeding methods and technologies for *Striga* resistance in maize

### 4.1 Conventional breeding methods

Plant breeding aims to develop genetically improved crop cultivars with economic benefits for small-scale and commercial farmers (Shimelis and Laing, 2012). This involves creating or expanding new genetic variations and selecting and fixing desirable genotypes in the progeny (Kumar and Shukla, 2014). The desired variation may be found in existing germplasms and artificially created through controlled crosses, induced mutations, and polyploidization. Appropriate methods and genetic analysis tools are required to detect and select the progeny that contains the target genotype to accomplish set breeding goals. Conventional, demand-led, and modern plant breeding methods are recent technologies used for trait introgression (Joshi, 2017). The development of new varieties through conventional breeding can take more than 10 years. However, integrating modern plant breeding methods [e.g., marker-assisted selection (MAS), quantitative trait loci (QTL) analysis, next-generation sequencing (NGS), and genome editing] can enhance variety development in a short time and at the lowest cost with a focus on performance and quality that meet the market requirement through the demand-led breeding approach (Matova et al., 2022).

### 4.2 Breeding methods in cross-pollinated crops

The main conventional breeding methods in cross-pollinated crops, such as maize, include mass selection, family selection, hybrid breeding, synthetic varieties, backcrossing, and recurrent selection. Hybrid breeding, backcrossing, and recurrent selection are the common breeding methods widely used in developing *Striga* tolerance/resistance maize varieties (Badu-Apraku et al., 2014).

#### 4.2.1 Hybrid breeding

Hybrid breeding involves crossing genetically complementary inbred lines to combine genes in desirable hybrids. It enables the development of new progenies and inbred lines with several traits (Anushma et al., 2021). The more divergent their parents are, the higher the heterosis in their hybrid or offspring (Reif et al., 2005). The main challenges in hybrid breeding are creating improved populations with desirable agronomic traits and local adaptation while keeping the populations genetically distant to explore hybrid vigor. Also, there is a need to develop efficient seed production of experimental hybrids (Flavio, 2013). Maize hybrids have been widely developed for *Striga* resistance and high yields. For instance, Menkir and Meseka (2019) reported maize hybrid series H01 to H16 (e.g., single crosses, three-way crosses, and double crosses) developed over three breeding periods that yielded 64% higher and supported 30% or less parasite emergence and damage than their checks (Gethi and Smith, 2004). Other *Striga* resistance in three-way-cross and single-cross hybrids were reported by Menkir et al. (2012) and were consistent against various *S. hermonthica* ecotypes in different regions. Some of the three-way hybrids were developed by crossing a common hybrid as a female parent with white grain inbred lines derived from ACRSYN-W and ZEADIPLO BC4 (Menkir et al., 2012). These hybrids are critical genetic resources for *Striga* resistance gene deployment programs through the breeding of hybrids and OPVs in the target production environments.

#### 4.2.2 Recurrent selection

Recurrent selection uses repeated cycles of selection and breeding aimed at accumulating useful genes for genetic improvement, especially traits conditioned by polygenes (White, 2004; Darrah et al., 2019). It is an ideal breeding approach to steadily improve quantitative traits in a breeding population. This technique has been widely used to improve maize for *Striga* resistance and economic traits (Badu-Apraku et al., 2007a; Menkir and Kling, 2007; Badu-Apraku et al., 2008). Menkir and Kling (2007) reported a *Striga*-resistant variety TZL COMP1-W developed using selfed progeny (S1 and S2 lines) or full-sib family selection schemes. The advanced cycle (C6) of that variety significantly outyielded the base population (C0) by 1628 kg ha^−1^ and sustained a yield loss of 36%, which was nearly half of the yield loss (70%) recorded in C0 (Menkir and Kling, 2007). The synthetic variety 2000 Syn EE-W derived from TZEE-W Pop STR C0 after a cycle of S1 family recurrent selection and the improved populations, TZEE-W Pop STR C3 and TZEE-Y Pop STR C3, are reported to be the highest yielding entries under *Striga*-infested and *Striga* free environments (Menkir and Kling, 2007). These results have shown that reccurent selection is an effective method for increasing the frequency of desirable genes in a population.

#### 4.2.3 Backcrossing

Backcrossing is a breeding method that is used to transfer one or a few desirable genes from a donor parent to an agronomically superior and elite recipient parent. It is a form of recurrent hybridization and selection by which an excellent characteristic is added to an otherwise desirable genetic background. The goal is to develop an ideotype containing the novel allele and all other essential traits in the recurrent parent. It works well when the variety to be improved is an inbred line and the trait to be introgressed is monogenic or oligogenic (Thomas et al., 2016). Badu-Apraku et al. (2007a) reported the introgression of *Striga* resistance into maize by backcrossing, generation of S1 progenies, selection of *Striga*-resistant S1 lines, and two cycles of recombination of the selected S1 lines under artificial *Striga* infestation. Akaogu et al. (2019) also reported an increase in *Striga*-resistant levels in an inbred line TZdEI 352 through backcrossing. The two maize populations, TZE-W Pop DT STR C4 and TZE-Y Pop DT STR C4, were developed using backcrossing, inbreeding, hybridization, and continuous selection. These are valuable *Striga*-resistant populations with outstanding yield under *Striga* infestation. The germplasms derived from backcrossing have been useful in developing *S. hermonthica-*resistant OPVs of maize (Badu-Apraku et al., 2007a).

#### 4.2.4 Doubled haploid technique

The development of homozygous and contrasting inbred lines are essential for maize breeding programs for hybrid or synthetic variety breeding with *Striga* resistance (Chaikam et al., 2019). The traditional inbred line development techniques requires six to eight generations of selfing and selection to achieve homozygosity. Conversely, the doubled haploid (DH) method enables the production of homozygote inbred lines from haploid individuals instantaneously (Trindade, 2022). The DH plants are derivatives of haploid plants with a doubled set of the chromosome. The DH method significantly speeds up the selection process by fixing genes in the homozygous state (Zargar et al., 2022). The DH method has several benefits, including creating lines with 100% homozygosity. Derived lines exhibit maximum genetic variability for selection and test crosses reducing breeding costs and increasing selection efficiency among experimental hybrids. Also, the DH technology exposes recessive and minor genes in homozygous backgrounds

**Figure 3:**
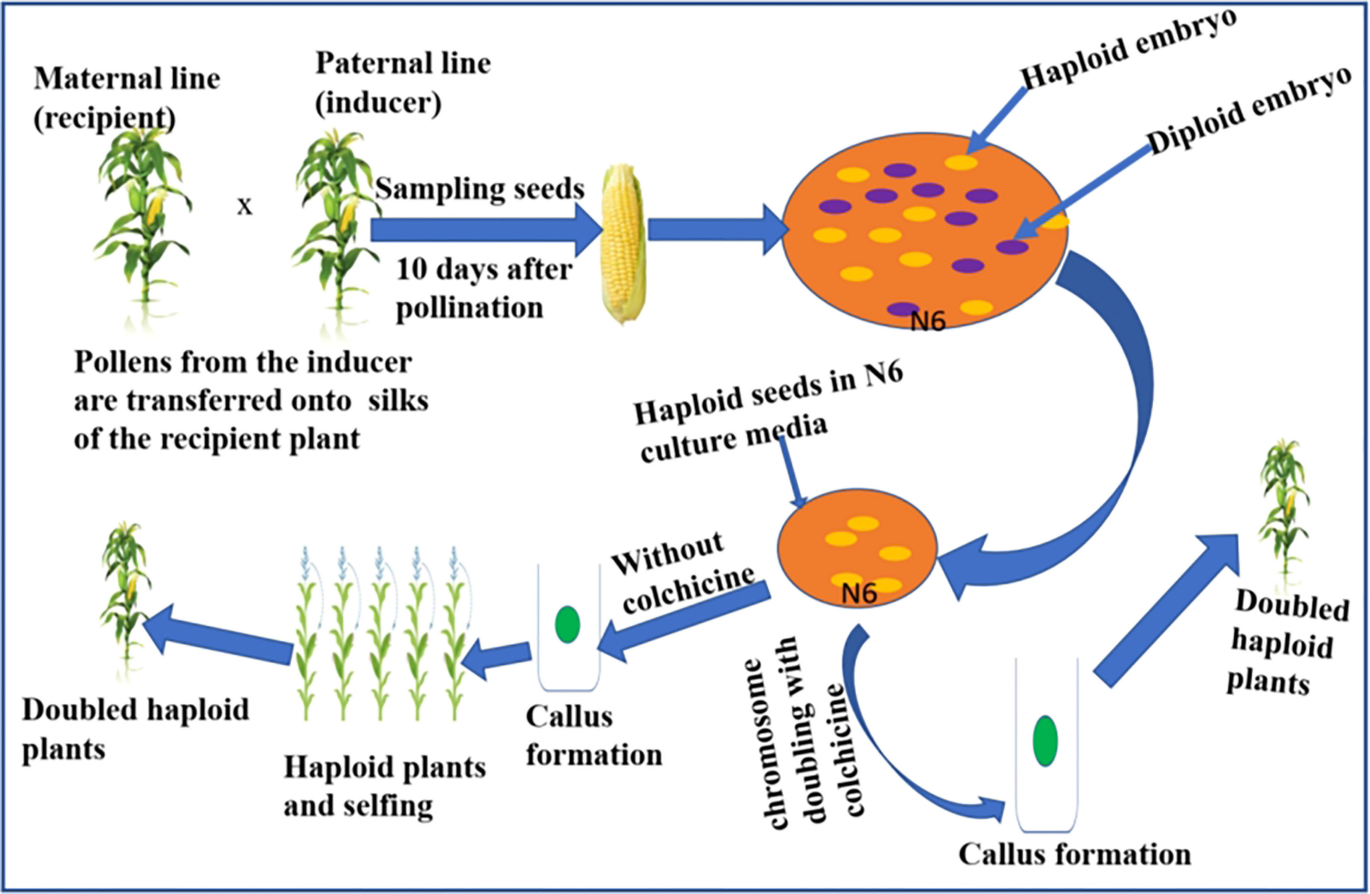
Scheme depicting the rapid development of DH lines in maize using inducer line. Note the fluorescent marker (haploid embryo) and the purple marker (diploid embryo). The embryos that lack purple color or with fluorescent signals are haploids and will be subjected to chromosome doubling in the culture media (N6) using colchicine.

DH lines are developed *in vivo* or *in vitro*. The *in vitro* method involves the use of haploid pollen or anther or female egg cells to induce haploid embryos. This is followed by chromosome doubling of the haploid individuals using chemical agents. The method has shown little success in reliably producing the large number of DH lines required for maize breeding programs (Chaikam et al., 2019; Mitiku, 2022). Hence, the method has yet to be standardized for maize breeding. The *in vivo* haploid induction has been successfully used in DH production in maize breeding programs (Mitiku, 2022). The *in vivo* procedures include haploidization following wide crosses and intra-specific crosses. Inter-generic or inter-specific crosses with cultivated maize genotype deliver haploid individuals, which are subjected to chromosome doubling to extract desirable DHs with agronomic traits and *Striga* resistance. During wide crosses, the chromosomes of the distantly related species are eliminated during early embryogenesis, leaving a haploid embryo (Ishii et al., 2016). Also, during intra-specific crosses, a selected maize parent can be crossed using pollen grains from a distant parent following pollen treatment with chemical or physical mutagenic agents followed by embryo rescue (Trentin, 2019). *Striga* resistance breeding has been attempted through maize-sorghum hybridization. However, the hybridization between sorghum and maize was only feasible when the crossing was supplemented with a 2, 4-D auxin hormone (Mueni, 2018). The most effective and quickest *in vivo* method to create DH lines is using a haploid inducer line. Crosses involving a standard maize genotype with a haploid inducer line generate haploid seeds enabling the development of DHs after treating tissue culture established seedling plants with chemical agents such as colchicine (Segui-Simarro et al., 2021). **Figure 3** summarizes a scheme showing a rapid method to develop haploids and DHs in maize using a haploid inducer line. Briefly, the technique involves pollination from the inducer line onto silks of the standard genotype, e.g. *Striga* resistance line. After pollination, the haploid embryos are rescued on a suitable culture media, followed by chromosome doubling using colchicine to develop DH lines. In the absence of colchicine treatment, the callus formed gives rise to haploid individuals that can be selfed to create DH plants.

Wilde et al. (2010) reported the effectiveness of the DH method enabling the exploitation of minor gene effects for low nitrogen adaptation in some European maize landraces. Maize breeders in the Water Efficient Maize for Africa (WEMA) project used the DH method to develop drought-tolerant inbred lines (Beyene et al., 2013). The authors reported that subsequent DH-derived hybrids outyielded commercial hybrid checks that were developed conventionally. Furthermore, the genetic variance of DH lines for GY, plant height, ear height, and leaf length was twice as high than the parental source of the S_1_ families.

The DH method can be successfully used in the selection of *Striga-*resistant maize germplasm for breeding. To produce DH resistant to *Striga*, the breeder should cross a female line resistant to *Striga* (recipient parent) with a male haploid inducer (donor parent). The seed of the F1 plants is sampled to differentiate haploid and diploid embryos. Only haploid embryos will be selected and transferred into a culture media. The haploid embryo can be cultured with a chromosome-doubling chemical reagent such as colchicine leading to callus formation and subsequently the DH plants, which are homozygous for the genes of interest, that is, *Striga* resistance. Alternatively, the haploid embryos can be cultured without any chemical reagent to developing haploid plants that could be selfed to select DH plants with *Striga* resistance. The DH approach will enhance the breeding efficiency for *Striga*-resistant hybrids or open-pollinated varieties. Furthermore, the use of the DH lines with effective molecular markers enables the identification of targeted QTLs linked to agronomic traits of importance at a lower cost (Mayor and Bernardo, 2009). Molecular markers associated with complex polygenic economic traits can be effectively discerned in DH populations. This will limit the number of genetic materials handled and assayed during phenotyping while increasing the frequency of favorable alleles (Prasanna, 2014). DH lines in tropical maize have been successfully developed using temperate haploid inducers (Prigge et al., 2011). In another study, Battistelli et al. (2013) reported the successful development of DH lines in tropical maize using Krasnodar Embryo Marker Synthetic (KEMS). Furthermore, Beyene et al. (2013) evaluated DH-derived testcross performance under drought stress and non-stress conditions. The authors reported significant differences in trait performance between the drought stress and well-watered conditions for all measured traits except for anthesis date. This suggests that DH derivatives perform better than genotypes developed through the conventional inbreeding platform owing to their homozygosity and genetic stability. Therefore, through DH technology, successful *Striga*-resistance breeding can be attained in maize

These studies suggest the possibility of successful *Striga*-resistant DH development in maize.

## 5 Phenotyping for *Striga* resistance

Until recently, conventional breeding was based on the phenotypic selection of target traits, which could be qualitative or quantitative (Hee-Jong et al., 2015). Phenotypic traits can be quickly and readily selected when the trait of interest is conditioned by major genes. This will yield easily distinguishable genotypes. In contrast, quantitative traits, including *Striga* resistance and yield-influencing traits, are challenging to select phenotypically due to the involvement of polygenes with minor genetic effect and their low heritability (Nazeer et al., 2010; Schuster, 2011; Araus and Cairns, 2014). The other limit of phenotypic selection in segregating populations is that only the traits conditioned by dominant genes are selected for the next round of crossing, because recessive genes are hidden and may be lost in advanced populations. Furthermore, the duration of selection cycles takes several years, which delays the development and release of new varieties. Therefore, many breeding programs have stagnated because of timelapse and the paucity of analytical tools for phenotyping and genetic analyses (Hee-Jong et al., 2015; Rincent et al., 2018). Recent advances in high-throughput phenotyping and genotyping have provided fast genetic information for accelerated breeding.

### 5.1 Stay green traits for *Striga* resistance in maize

The damage inflicted by *Striga* is through the extraction of the host’s metabolites and the secretion of phytotoxic compounds that leave the host with a diseased-like appearance. The stay green (SG) genotype selection may be an essential component of *Striga* resistance in maize. Genotypes with SG character have the capacity to maintain their leaves photosynthetically active. This will subsequently improve grain development and filling, including under stress conditions (Vadez et al., 2013).

Kamal et al. (2019) reported functional and non-functional SG genotypes. The functional SG genotypes maintain their photosynthetic capacity compared with the non-SG genotypes. Functional SG genotypes delay the onset of senescence, or the senescence syndrome is initiated and grows slowly (Thomas and Ougham, 2014). Conversely, in the non-functional SG genotypes, senescence is commenced on an average time scale; however, leaf greenness is extended because of the failure of the chlorophyll degradation pathway, with a decline in photosynthetic capacity (Kamal et al., 2019). Both forms of SG may be associated with *Striga* resistance and GY improvement in maize.

Also, SG has been identified as an important component of the genetic improvement of several crops. For instance, Luche et al. (2013) reported the association between SG and GY improvement in wheat, while Silva et al. (2004) reported the association between SG and higher grain quality in wheat. The association between SG with tolerance to abiotic and biotic stresses has also been reported by Kassahun et al. (2009) and Joshi et al. (2006) in sorghum and wheat, respectively. SG has been extensively used to improve tolerance to drought and heat in sorghum (Sanchez et al., 2002; Vadez et al., 2013) and maize (Wang et al., 2012; Cerrudo et al., 2017). There is ongoing research at the International Crops Research Institute for the Semi-Arid Tropics (ICRISAT) to transfer QTLs conditioning SG into genetically diverse, tropically adapted elite sorghum varieties for drought tolerance through marker-assisted backcrossing (MABC) (Isaac et al., 2019). Yang et al. (2017) studied SG QTLs in maize using a heterogeneous inbred family approach and found that the SG-associated parameters were significantly correlated with crop yield. These QTLs could be targeted for marker-assisted backcross transfer into maize varieties to improve maize yield under *Striga* infestation.

### 5.2 High-throughput phenotyping techniques for *Striga* resistance

High-throughput phenotyping platforms (HTPP) have revolutionized phenomics in plant breeding programs in the last 10 years (Jangra et al., 2021). HTPP relies on high data recording capacities, data collection and analysis speed, and non-destructive, non-invasive, and remote-sensing phenotyping techniques (Prasanna et al., 2013). Its utilization allows for the screening of many plants at various phenological stages so that desired traits can be rapidly screened at the initial stages, eliminating the need to wait for plant maturation in the field (Cabrera-Bosquet et al., 2012). HTPP can be used in the laboratory and field. The following platforms are some examples of HTPP used in cereal breeding programs: the ScanAlyzer HTPP platform developed by LemnaTec (Virlet et al., 2016), PHENOPSIS developed by Optimalog, on contract by the Laboratory of Plant Ecophysiological responses to Environmental stresses, in Montpellier, France (Granier et al., 2006), and DuPont-Pioneer’s FAST Corn (Jin et al., 2021).

Near-infrared spectroscopy (NIRS) is a chemometric technique that combines spectroscopy and mathematics to rapidly produce indirect, quantitative estimates of the concentration of organic compounds. This technique has been widely implemented in laboratory analysis of grain traits in several crops, such as maize, sorghum, and wheat, due to its low cost, rapidity, high precision, and repeatability for assessing several plant traits (Jangra et al., 2021). Imaging Technologies for Plant Phenotyping, Visible Light Imaging, and Fluorescence are also high-throughput phenomic platforms that have emerged as promising methods for the high-throughput phenotyping of crops (Das et al., 2015; Knecht et al., 2016; Jangra et al., 2021). Quality and yield, spikelet number, main orthogonal grain dimensions, maize cob, ear, and kernel attributes have been studied using the technology of structural imaging (Igathinathane et al., 2009; Miller et al., 2017; Makanza et al., 2018). All these platforms are opportunities to be explored to speed up phenotyping for *Striga* resistance in maize. Using high-throughput genotyping (HTG) in combination with high-throughput digital phenotyping, it is possible to increase marker density and improve the precision and resolution of QTL detection (Bhat et al., 2020).

### 5.3 Selection using biochemical markers

Genetic markers can be grouped into two categories: classical markers, including biochemical markers, and DNA/molecular markers. Biochemical markers or isozymes are multi-molecular forms of enzymes that are coded by various genes but have the same functions (Bailey, 1983). Biochemical markers have been successfully applied in the detection of genetic diversity, population structure, gene flow, and population subdivision (Nadeem et al., 2017). Abdellatif and Khidr (2010) examined the genetic diversity of new maize hybrids based on simple sequence repeat (SSR) markers and biochemical markers and concluded that both molecular (e.g., SSR, ISSR, and RAPD) and biochemical markers (e.g., seed storage protein content) are efficient tools. Rahim (2019) reported significant variation among maize accessions using morpho-biochemical markers. Biochemical markers are easy to use and cost effective. However, they are fewer in number and detect less polymorphism (Mondini et al., 2009; Nadeem et al., 2017).

*Striga* seed germination requires the detection of SL, a group of chemical compounds released from host plants’ roots (Mishra et al., 2016). After the *Striga* seed germination, the radicle of the germinating seed penetrates the host root, subsequently forming a haustorium to establish a xylem–xylem connection with the host, through which to withdraw water and nutrients (Jamil et al., 2011). Maize plants that release high levels of SL promote higher levels of *Striga* infestation (Jamil et al., 2011; Jamil et al., 2012). Hence, maize varieties with low levels of SL secretion should have reduced levels of *Striga* infestation, thereby reducing maize yield losses in the field under *Striga* infestation. Quantification of the release of SL could serve as biochemical markers to select for *Striga* resistance maize genotypes.

## 6 Genomic-assisted techniques to accelerate *Striga* resistance breeding in maize

### 6.1 Marker-assisted selection

MAS is the process of using morphological, biochemical, or DNA markers to select plants carrying genomic regions involved in expressing phenotypic traits of interest (Choudhary et al., 2008). Molecular markers, genetic maps, and associated low-cost genotyping technologies powered by advances in genomic sequencing and the advent of single-nucleotide polymorphism (SNP) markers have facilitated MAS for qualitative and quantitative traits (Boopathi, 2020; Rajesh et al., 2021). The selection can occur at different stages in a maize breeding program. The first possible stage is when selecting elite lines to create inbred lines (Ragot et al., 2007). At that stage, markers are used to distinguish heterotic groups from which inbred lines are developed by crossing elite lines within heterotic groups and hybrids with high heterosis are developed by crossing lines from different heterotic groups (Semagn et al., 2012). Several studies have been conducted to determine *Striga-*resistant heterotic groups. For example, Menkir et al. (2005) identified heterotic groups of *Striga*-resistant inbred lines among source populations using amplified fragment length polymorphism (AFLP) and SSR markers. Mengesha et al. (2017) reported four heterotic groups of *Striga*-resistant inbred lines using SNPs. These inbred lines are available for *Striga*-resistant breeding programs.

The most direct use of MAS in maize is MABC where targeted genes are introgressed into elite inbred lines (Crosbie et al., 2006). MABC has been implemented to improve traits in maize and other cereal crops. For example, Ribaut and Ragot (2007) used MABC to improve drought adaptation in maize varieties. Yohannes et al. (2015); Ali et al. (2016), and Ngugi et al. (2015) reported MABC for *Striga* resistance in sorghum. However, no study has been yet reported on MABC in *Striga* resistance in maize. Marker-assisted recurrent selection (MARS) is another commonly used method to increase the frequency of favorable alleles in crops by enhancing the efficiency of recurrent selection and accelerate the progress of the procedure (Kebede et al., 2021). Beyene et al. (2016) reported the use of MARS to improve maize GY under drought stress by crossing two elite *Striga*-resistant inbred lines (Acr.SynW-S2-173-B∗ 4) and (TZLComp.1C4-S1-37-5-B∗ 3), which are also tolerant to drought. The authors reported GY increased from the original to the advanced selection occurred only under drought stress (Abdulmalik et al., 2017). SNP markers linked to *Striga* resistance in maize have been widely discovered. SNPs have become the markers of choice due to their low cost per data point, high genomic abundance, locus specificity, co-dominance, the potential for high-throughput analysis, and lower genotyping error rates (Lipka et al., 2012; Adu et al., 2019). The SNPs reported by Stanley et al. (2021); Mengesha et al. (2017), (Pfunye et al., 2021) suggest the possibility of successful MAS in the future.

Integrating MAS with conventional breeding techniques will accelerate breeding and genetic gain. A persistent problem with the use of discrete genomic markers is that their presence in a genome does not guarantee their phenotypic expression, which is particularly important with polygenic, quantitative traits, where multiple SNPs are involved in the expression of a single trait, such as *Striga* resistance.

### 6.2 Quantitative traits loci analysis

Genomic tools such as molecular markers help identify, locate, and mapping genes responsible for economic traits such as *Striga* resistance and agronomic traits in maize. The technology involved linkage mapping and association or linkage disequilibrium (LD) mapping. Genetic recombination is the basis for linkage and association mapping (Wang and Qin, 2017). Linkage mapping is traditionally used based on genetic recombination events during the development of populations (Sonah et al., 2015). Mapping populations developed through initial biparental crosses lead to the development of F2, backcrosses [BCs], recombinant inbred lines [RILs], DHs, and near-isogenic lines [NILs], with a small number of accumulated recombination events (Balasubramanian et al., 2009). The QTL intervals found are extended over several centimorgans (cMs), and a genetic distance translates into large genomic regions with many candidate genes. This renders linkage mapping with a relatively low-mapping resolution and allele richness (Xu et al., 2017). Conversely, association mapping is a complementary tool to linkage mapping that resolves complex trait variation by exploiting historical and evolutionary recombination events at the population level (Xu and Crouch, 2008; Myles et al., 2009).

With the advent of rapid genome-wide, high-density marker data using high-throughput and NGS technologies, genome-wide association studies (GWAS) have become a standard tool for identifying resistance genes and loci (Adewale et al., 2020). GWAS, also known as whole-genome association study (WGAS), identifies genomic regions controlling quantitative economic traits (Adewale et al., 2020). It includes collecting a sample population, phenotyping, genotyping, and testing the association between phenotypes and genotypes using statistical approaches. GWAS is used to evaluate the association between each genotyped marker and a phenotype of interest that has been scored across a large number of individuals to guide MAS (Korte and Farlow, 2013).

However, GWAS has some limitations in identifying unknown genes (Lipka et al., 2012). The first limitation is the diversity panels of crop species that often represent a strong population structure, leading to false positives because of spurious associations between the phenotypes and unlinked markers (Benavente and Giménez, 2021; Phuke et al., 2022). The second limitation is the large extent of LD, which often ranges over several hundred kilobases. This may result in the inclusion of many candidate genes in a single LD block exhibiting a significant signal, thus entailing the need for additional experiments to conclusively identify the causal gene(s) (Yano et al., 2016). A nested association mapping population (NAM), a multiparent advanced generation intercrossing population (MAGIC), and random-open-parent association mapping (ROAM) are suitable for GWAS, because they are enriched in variations and have inconspicuous population structures that are more powerful for QTL mapping (Cavanagh et al., 2008; Tian et al., 2011; Huang et al., 2021). The NAM approach provides high-genetic variation while avoiding the complications of genetic structure, whereas MAGIC can examine the effect of loci, unbiased due to the balanced contributions from all founders (Wang and Qin, 2017). ROAM improves genetic resolution and statistical power to identify variants with minor effects and low frequency (Fang and Luo, 2019).

With a well-defined population and an appropriate statistical model, GWAS is among the most prominent and reliable approaches for detecting the whole genetic architecture of a trait. **Table 5** summarizes to date candidate genes/QTLs conferring resistance to *Striga*. The first QTLs associated with *Striga* resistance in maize were reported by Amusan (2010). Two QTLs were mapped on Chromosome 6 in the populations derived from an F2-mapping population involving a cross between the susceptible maize inbred lines 5057 and the resistant ZD05. These two QTLs accounted for 55% of observed phenotype variation for incompatible responses to *S. hermonthica* infestation on host roots. The second report was in a study conducted by Adewale et al. (2020) using GWAS. The locus S9_154,978,426 on Chromosome 9 was found at 2.61 Mb close to the ZmCCD1 gene, which is known to be associated with reducing SL production in the maize roots (Adewale et al., 2020). Badu-Apraku et al. (2020a) identified 14 QTL associated with *S. hermonthica* resistance indicator traits, comprising three QTLs for GY, four QTLs for ear per plant, and seven QTLs for *S. hermonthica* damage, at 10 weeks after planting across environments, and found 154 candidate genes associated with *Striga* resistance/tolerance traits. Another 12 QTLs associated with *S. hermonthica* resistance traits were found in an F2:3 population involving a cross between yellow inbred lines, TZEEI 79 (*Striga* resistant/tolerant) and TZdEEI 11 (*Striga* susceptible) by Badu-Apraku et al. (2020c) using QTL mapping. Pfunye et al. (2021) identified three SNPs on Chromosomes 5, 6, and 7 linked to the total *Striga* plant emergence. Gowda et al. (2021) reported 57 SNPs significantly associated with *Striga* resistance indicator traits and GY under artificial *Striga* infestation with low to moderate effect. They found 32 candidate genes physically near the significant SNPs with roles in plant defense against biotic stresses.

**Table 5:** Trait-specific genes/QTL in maize conferring *Striga* resistance.

Genes/QTL	Chromosome	Traits associated	Study approach	Reference
**GRMZM2G408305**	1	Grain yield	QTL mapping	Badu-Apraku et al. (2020a)
**GRMZM2G324999**	1	Ear per plant		
**GRMZM2G174784**	2	Ear per plant		
**Zma-MIR167 g**	3	Ear per plant		
**GRMZM2G053503**	8	Ear per plant		
**GRMZM2G059851**	5	*Striga* damage rating		
**GRMZM6G199466**	7	Grain yield, ears per plant, and *Striga* damage		
**GRMZM2G044194**	7	Grain yield, ears per plant, and *Striga* damage		
**GRMZM2G008234**	7	Grain yield, ears per plant, and *Striga* damage		
**GRMZM2G054050**	3	*Striga* emergence count		
**GRMZM2G085113**	3	Grain yield	QTL mapping	Badu-Apraku et al. (2020c)
**GRMZM2G050550**	7	Grain yield		
**GRMZM2G027563**	7	Ears per plant		
**GRMZM2G143204**	1	*Striga* damage		
**GRMZM5G803355**	3	*Striga* damage		
**GRMZM2G045431**	7	*Striga* damage		
**GRMZM2G113060**	9	*Striga* damage		
**GRMZM2G301485**	10	Grain yield, *Striga* damage		
**GRMZM2G030762**	8	Ears per plant, *Striga* damage		
**GRMZM2G180328**	6	Ears per plant, *Striga* damage		
**GRMZM2G164743**	10	Grain yield, *Striga* damage	GWAS	Adewale et al. (2020)
**GRMZM2G080044**	9	Grain yield		
**GRMZM5G898880**	9	Grain yield		
**GRMZM2G057243**	9	*Striga* damage		
**GRMZM2G060216**	3	*Striga* damage		
**GRMZM2G310674**	10	Ears per plant		
**GRMZM2G016836**	7	Ears per plant		
**GRMZM2G315127**	5	Ears per plant		
**EREB139, GRMZM2G103085**	5	Ears per plant		
**GRMZM2G018508**	5	*Striga* emergence count	GWAS	Gowda et al. (2021)
**GRMZM2G015520**	7	*Striga* damage		
**GRMZM2G143086**	1	*Striga* damage		
**GRMZM2G422670**	1	*Striga* damage		
**GRMZM2G094771**	3	Grain yield		
**GRMZM2G023051**	6	Grain yield		
**GRMZM2G157836** **GRMZM5G881641**	4	Grain yield	GWAS	Stanley et al. (2021)
**GRMZM2G406758** **GRMZM2G110289**	9	Grain yield		
**GRMZM2G180262**	10	*Striga* damage		
**GRMZM2G024099**	1	*Striga* damage		
**GRMZM2G351582**	1	*Striga* damage		
**GRMZM2G028521**	1	*Striga* damage		
**GRMZM2G092128**	2	*Striga* damage		
**GRMZM2G102242**	2	*Striga* damage		
**GRMZM2G414252**	2	*Striga* damage		
**GRMZM2G171830**	2	*Striga* damage		
**GRMZM2G162781**	2	*Striga* damage		
**GRMZM2G081285**	4	*Striga* damage		
**GRMZM2G112548**	5	*Striga* damage		
**GRMZM2G113418**	5	*Striga* damage		
**GRMZM2G035073**	5	*Striga* damage		
**GRMZM5G832409**	6	*Striga* damage		
**GRMZM2G162382**	7	*Striga* damage		
**GRMZM2G300965 GRMZM2G300969**	10	*Striga* damage		
**GRMZM5G873586 GRMZM2G356817**	10	*Striga* damage		
**GRMZM2G364748**	10	*Striga* damage		
**GRMZM2G017470**	1	*Striga* emergence count		
**GRMZM2G088778**	2	*Striga* emergence count		
**GRMZM2G179505**	2	*Striga* emergence count		
**GRMZM2G701566**	3	*Striga* emergence count		
**GRMZM2G129543**	5	*Striga* emergence count		
**GRMZM5G823157**	5	*Striga* emergence count		
**GRMZM2G033413**	9	*Striga* emergence count		
**GRMZM2G006948**	9	*Striga* emergence count		
**GRMZM2G063575**	10	*Striga* emergence count		

Few studies have reported on QTLs and genes linked to *Striga* resistance in maize. Using GWAS, Okunlola et al. (2023) identified 22 SNP markers significantly associated with GY and *Striga* damage rating at 10 weeks after planting, the number of emerged *Striga* plants at 8 and 10 weeks after planting, and ear aspect. There are several SNPs and QTLs involved in *Striga* resistance. Hence, the breeder needs to filter useful markers for MAS.

### 6.3 Next-generation sequencing

The development and application of molecular markers in crop genetics have gained remarkable success in the past three decades. The use of molecular markers in crop improvement programs started from low-throughput morphological markers (Soltabayeva et al., 2021), restriction fragment length polymorphism (RFLP) (Powell et al., 1996; Ahmar et al., 2020) to SNP markers based on NGS (Jenkins and Gibson, 2002; De Donato et al., 2013). Maize populations have been genotyped using several methods, such as Random Amplified Polymorphic DNA (RAPD) (Carvalho et al., 2004), RFLP (Garcia et al., 2004), and AFLP (Schrag et al., 2006). The SNPs are the most widely used markers for assessing the genetic diversity and association mapping in maize (Dos Santos et al., 2016; Kasoma et al., 2020; Tomkowiak et al., 2021). The SNPs can be observed through various experimental protocols.

Genotyping by sequencing (GBS) (De Donato et al., 2013), restriction-associated DNA (RAD), complexity reduction of polymorphic sequences (CRoPS) (van Orsouw et al., 2007), and diversity arrays technology (DArT) are among available approaches to do SNPs discovering and genotyping. However, GBS is the most popular approach to the identification of SNPs in plants (Negro et al., 2019; Heinrich et al., 2020; Lee et al., 2021). The GBS is a novel application of NGS protocols for discovering and genotyping SNPs in crop genomes and populations. The development of NGS technologies enhanced the genomics landscape in crop breeding. Several NGS technologies such as Roche 454 FLX Titanium, Illumina, MiSeq, and HiSeq 2500 have been developed. These technologies provide various genotyping platforms with great improvements in coverage, time, and costs (Bevan et al., 2017).

The DArT is a sequence-independent, high-throughput, reproducible, cost-effective, and whole (Jaccoud et al., 2001) genome genotyping technology. The DArT method allows simultaneous detection of several thousand DNA polymorphisms (depending on the species) arising from single-base changes and small insertions and deletions (InDels). The process involves scoring the presence or absence of DNA fragments in genomic representations generated from genomic DNA samples through a process of complexity reduction (Jaccoud et al., 2001). This is achieved using a combination of restriction enzymes that separate low-copy sequences (most informative for marker discovery and typing) from the repetitive fraction of the genome. The combination of the complexity reduction of the DArT method with NGS led to DArTseq technology for identifying DArT markers through the DArTseq protocol (Kilian et al., 2012; Raman et al., 2014). DArTseq is the most preferred method of GBS (Sanchez-Sevilla et al., 2015). The initial DArT implementation on the microarray platform involved fluorescent labeling of representations and hybridization to dedicated DArT arrays. The DArTseq method deploys sequencing of the representations on the NGS platforms (https://www.diversityarrays.com/technology-and-resources/dartseq/). Most recent studies on genotyping maize for *Striga* resistance used the DArTseq method (Adewale et al., 2020; Badu-Apraku et al., 2020a; Badu-Apraku et al., 2020c; Yacoubou et al., 2021c; Zebire et al., 2021). However, more reliable markers linked to *Striga* resistance in maize should be explored to facilitate breeding for *Striga*-resistant maize through MAS.

### 6.4 Genetic engineering and genome editing technologies

#### 6.4.1 Genetic engineering

Abiotic and biotic stress, including *Striga* damage, cause an extensive loss to agricultural production and productivity worldwide (Gull et al., 2019). A combination of breeding approaches and new technologies could significantly improve crop stress resistance/tolerance in the field. Genetic engineering, also called genetic modification (GM), uses laboratory-based technologies to produce a desired trait by adding a gene from one species to an organism from a similar or different species (Mackelprang and Lemaux, 2020; Sahu, 2020). GM of crop plants has been achieved for GY and other economic traits improvement. For example, herbicide-resistant maize and soybean and Bt-derived maize, soybean, and cotton are some of the genetically engineered crops developed to date (Mittler and Blumwald, 2010; Basnet et al., 2022). Several studies, including meta-analyses, have reported the economic potential of genetic engineering crops to harness crop productivity (Areal et al., 2012; Klumper and Qaim, 2014; Pellegrino et al., 2018). There are increasing cases that GM products are safe for human consumption and the environment (Fernbach et al., 2019). However, the risks and benefits of GMOs have led to fears that are still being debated (Blagoevska et al., 2021).

#### 6.4.2 Genome editing

Genome editing (GE) is a suite of advanced biotechnological techniques that allows for the precise and efficient modification of an organism’s genome. The technology includes mega-nucleases or homing endonucleases (HEs), zinc finger nucleases (ZFNs), transcription activator-like effector nucleases (TALENs), and Type II clustered regularly interspaced short palindromic repeat (CRISPR)/CRISPR-associated protein (Cas) (Hua et al., 2019; Ku and Ha, 2020; Trindade, 2022). These GE systems generate targeted DNA double-strand breaks (DSBs) in the genome. The potential of CRISPR in maize improvement was reported by various authors. Feng et al. (2016) and Zhu et al. (2016) reported efficient target genome modification in maize using the CRISPR/Cas9 system. More recently, aromatic maize has been created using CRISPR/Cas9 (Wang et al., 2021). However, no study has reported on the use of genetic editing for *Striga* resistance improvement in maize.

The above techniques are promising and offer new opportunities for developing new *Striga*-resistant maize varieties in the shortest time possible. The availability of *Striga* resistance genes in maize will facilitate the use of genetic engineering and gene editing for *Striga*-resistant maize development. Therefore, there is a need to identify more *Striga*-resistant genes or QTLs that confer resistance to maize. The development of high-throughput DNA-sequencing technology and the establishment of a large number of “omics” databases will facilitate the identification of useful *Striga* resistance traits for genome editing in maize.

## 7 Conclusion and outlook

The parasitic weed *Striga* has a devastating impact on cereals, especially maize production in SSA. Many organizations have been involved in developing strategies to combat the parasite; thus, several control methods have been developed and tested. This has led to the development of a range of genetic resources of maize existing for *Striga* resistance. Several maize varieties with partial resistance have been developed and released in Africa. However, no best practice is available to control the *Striga* parasite in maize, needing integrated management spearheaded by *Striga*-resistance breeding. Primary genetic resources of maize for *Striga* resistance, including landraces, wild relatives, mutants, and synthetic varieties, reported in this paper may guide new variety designs in maize for *Striga*-resistance and desirable product profiles. Integrating conventional breeding, mutation breeding, and genomic-assisted breeding (i.e., MAS, QTL analysis, NGS, and genome editing) will enhance genetic gains in *Striga* resistance breeding programs.

## Author contributions

ED: Conceptualization; Investigation; Methodology; Resources; Writing original draft; Writing – Review & Editing. HS: Conceptualization; Funding acquisition; Methodology; Project administration; Resources; Supervision; Validation; Visualization; Writing – Review & Editing. EM: Validation; Visualization; Writing – Review & Editing. AS: Validation; Visualization; Writing – Review & Editing. ML: Funding acquisition; Resources; Supervision; Validation; Visualization; Writing – Review & Editing.
