# Erratum: Genetic resources and breeding of maize for *Striga* resistance: a review

**DOI:** 10.3389/fpls.2023.1254773

**Published:** 2023-07-21

**Authors:** Frontiers Production Office

**Affiliations:** Frontiers Media SA, Lausanne, Switzerland

**Keywords:** doubled haploid, genetic resources, gene editing, genomic resources, maize breeding, quantitative traits loci, *Striga* species

Due to a production error, **Table 1** was formatted incorrectly as published. A row reference was incorrectly placed within a column.

The corrected **Table 1** appears below:

**Table 1:** Some genetic sources of *Striga* resistance in maize, sorghum, pearl millet, and rice.

Crops	Type of variety	Name or designation	*Striga* reaction	Unique traits	Country and reporting organization	References
Maize	Wild relative	*Tripsacum dactyloides*	Pre-attachment resistant	Inhibition of haustorial development	IITA, Nigeria	Gurney et al. (2003)
		*Zea diploperenis*	Post-attachment resistant	Barrier development after haustorial development		Amusan et al. (2008)
	Line	ZD05	Post-attachment resistant	Low level of *Striga* attachment and high mortality of attached parasites		
		TZdEEI 7	Post-attachment resistant	Barrier development after haustorial development		Shaibu et al. (2021)
		TZEEI 63				
		TZdEEI 1				
	Landraces	CRIC 51	Pre-attachment resistant	Low level of *Striga* germination	CIMMYT, Kenya IITA, Nigeria, KARI, Kenya	Karaya et al. (2012)
		VERA 217				
		CUBA T-31				
		BRAZ 1758				
		BRAZ 1279				
		CRIC 51				
		Mochore	Pre-attachment resistant	Low level of *Striga* germination	ICIPE, Kenya	Midega et al. (2016)
		Nyamula				
		Sefensi				
		Jowi				
Sorghum	Wild relatives	*Sorghum versicolor*	Post-attachement resistant	Hypersensitivity	IACR-Long Ashton Research Station	Haussmann et al. (2000)
		*Sorghum drummondii*	Pre-attachment resistant	Low haustorium initiation		Ramaiah (1986)
	Lines	SRN 39	Post-attachment resistant	Low production of the germination stimulant	ICRISAT, Burkina-Faso	
		IS 9830	Post-attachment resistant	Low production of the germination stimulant		
		IS 15401	Post-attachment resistant	Low production of the germination stimulant		
		SAR 16	Post-attachment resistant	Low production of the germination stimulant, hypersensitivity		
		SAR 19	Post-attachment resistant	Low production of the germination stimulant, hypersensitivity		
		SAR 33	Post-attachment resistant	Low production of the germination stimulant, hypersensitivity		
	Cultivars	N 13	Post-attachment resistant	Mechanical barriers, antibiosis	ICRISAT, Mali	Gurney et al. (2002), Haussmann et al. (2000)
		Framida	Post-attachment resistant	Mechanical barriers	ICRISAT, Mali	
Pearl millet	Wild accessions	*PS 202*, PS 637, PS 639, PS 727	Pre-attachment resistant	Low level of *Striga* attachement	ICRISAT, Mali	Wilson et al. (2000)
	Landraces	M141, M239, M029, M197, M017 and KBH	Pre-attachment resistant	Lower level of *Striga* attachment, lower downy mildew incidence, higher panicle yield	IRD, France ICRISAT, Niger	Kountche et al. (2013)
Rice	Cultivars	Nipponbare	Post-attachment resistant	Absence of parasite–host xylem–xylem connections	IRRI, Philippines	Gurney et al. (2006)

IRD, Institute for Research Development/France; ICRISAT, International Crops Research Institute for the Semi-Arid Tropics/India. IRRI, International Rice Research Institute/Philippines; IACR, Institute for Arable Crops Research/India; IITA, International Institute of Tropical Agriculture/Nigeria; KARI, Kenya Agricultural Research Institute; ICIPE, International Centre of Insect Physiology and Ecology/Kenya.

The publisher apologizes for this mistake.

The original version of this article has been updated.

